# Activin receptor-like kinase 4 haplodeficiency alleviates the cardiac inflammation and pacing-induced ventricular arrhythmias after myocardial infarction

**DOI:** 10.18632/aging.203236

**Published:** 2021-07-01

**Authors:** Yuli Yang, Qian Wang, Xingxing Cai, Zhixing Wei, Jianwen Hou, Yudong Fei, Wei Li, Yigang Li

**Affiliations:** 1 Department of Cardiology, Xinhua Hospital Affiliated to Shanghai Jiaotong University School of Medicine, Shanghai, China; 2 Department of Cardiology, The Second Affiliated Hospital of Zhejiang University School of Medicine, Hangzhou, Zhejiang, China

**Keywords:** ALK4, myocardial infarction, inflammation, macrophage, ventricular arrhythmia

## Abstract

Background: Inflammation process is an important determinant for subsequent changes in cardiac function and remodeling after acute myocardial infarction (MI). Recent studies have implicated that ALK4 haplodeficiency improves cardiac function after MI. However, it remains unknown if the beneficial effects are partly attributed to ALK4 haplodeficiency-induced modulation on inflammatory response in the inflammatory phase of MI. In this research, we aimed to explore the mechanism of ALK4 haplodeficiency in the inflammatory stage of MI.

Methods: ALK4, CD16, and CD14 were detected in peripheral blood mononuclear cells (PBMCs) isolated from MI patients and healthy volunteers. ALK4 haplodeficiency (ALK4^+/-^) mice and wild-type (WT) littermates were randomly divided into the sham group and the MI group. Inflammation cytokines and chemokines were measured. Echocardiography and intracardiac electrophysiological recordings were performed on the 3^rd^ day and the 7^th^ day after MI operation. ALK4 expression and inflammation cytokines were also detected in LPS- or IL-4-stimulated bone marrow-derived macrophages (BMDM) from the ALK4^+/-^ mice and WT littermates.

Results: ALK4 gene expression in circulating monocytes of MI patients was higher than that in those of healthy volunteers. Cardiac inflammation and vulnerability of ventricular arrhythmia after acute myocardial injury are significantly alleviated in ALK4^+/-^ mice as compared to WT littermates. On the 3^rd^ day post-MI, the level of M1 macrophages were decreased in ALK4^+/-^ mice as compared to WT littermates, while the level of M2 macrophages were increased on the 7^th^ day post-MI. BMDM isolated from ALK4^+/-^ mice displayed reduced secretion of pro-inflammation cytokines after stimulation by LPS in hypoxic condition and increased secretion of anti-inflammation cytokines after stimulation by IL-4. As a result, the haplodeficiency of ALK4 might be responsible for reduced inflammation response in the post-MI stage.

Conclusions: ALK4 haplodeficiency reduces cardiac inflammation, improves cardiac function, and finally reduces the vulnerability of ventricular arrhythmia in the inflammatory stage after MI.

## INTRODUCTION

Myocardial infarction (MI) is a leading cause of cardiac mortality and morbidity in the world [1–3]. Repair of the infarcted myocardium is divided into three overlapping phases, namely the inflammatory phase, the proliferative phase, and the maturation phase. In the infarcted heart, innate immune response pathways can be activated as the result of acute myocardial injury and further trigger the extensive inflammatory response which is necessary for the repair of the infarcted heart. The inflammatory response is also associated with the pathogenesis of adverse remodeling and consequently cardiac failure after MI. The timely inhibition of pro-inflammatory response is important for the healing of infarcted heart effectively. Cardiac remodeling after MI is a process accompanied by the alterations of cardiac geometry, function, and structure. A large number of endogenous factors including cytokines and matrix metalloproteinases and tissue inhibitors of matrix metalloproteinases affect the accumulation of extracellular collagen matrix and finally take part in the process of cardiac remodeling in different ways [4–6].

In the early phase of MI, macrophages are the main cells of the innate immune system. They can secrete cytokines and chemokines that play an important role in the pathological process after MI. When MI occurs, besides the cardiac resident macrophages, the monocytes deriving from the blood become another source of macrophages in the injured heart [7–10]. Macrophages deriving from monocytes and resident macrophages can both produce pro-inflammatory and anti-inflammatory factors to take part in the inflammatory response, promoting the absorption of cellular debris, regulating the formation of granulation tissue and neovascularization [11, 12]. Macrophages can be classified into different subtypes that exert different functions. M1 macrophages secrete large amounts of pro-inflammatory cytokines which can promote the development of inflammation and the accumulation of extracellular matrix. M2 macrophages are demonstrated to have immune-regulatory and anti-inflammatory properties, which contribute to the tissue remodeling, the formation of neovascularization, and the progression of tumors [12–14].

Recent studies implicate that activin A could promote the transformation of macrophages into proinflammatory phenotype [15]. ALK4, as the receptor of activin A, its haplodeficieny alleviates myocardial infarction-induced cardiac fibrosis and improves cardiac function on the 28^th^ day after MI [16]. However, it is still unclear if the beneficial effect is partly attributed to ALK4 haplodeficiency-induced modulation on the inflammatory process in the inflammatory stage of MI.

In our study, we explored the role of ALK4 in the inflammatory stage of MI-induced inflammation response. We found that ALK4 was upregulated in the heart tissues and macrophages on the 3^rd^ day post-MI. ALK4^+/-^ mice showed significant alleviation of inflammatory response in the infarct border zone and enhanced cardiac function on the 3^rd^ day post-MI. At the same time, more M2 macrophages were found transferred in the infarct zone on the 7^th^ day after MI. Taken together, our results indicated that targeting ALK4 might be a promising treatment option for modulating the inflammation process in the inflammatory phase of MI.

## RESULTS

### Upregulated ALK4 expression in circulating monocytes of patients with acute myocardial infarction

**Figure 1:**
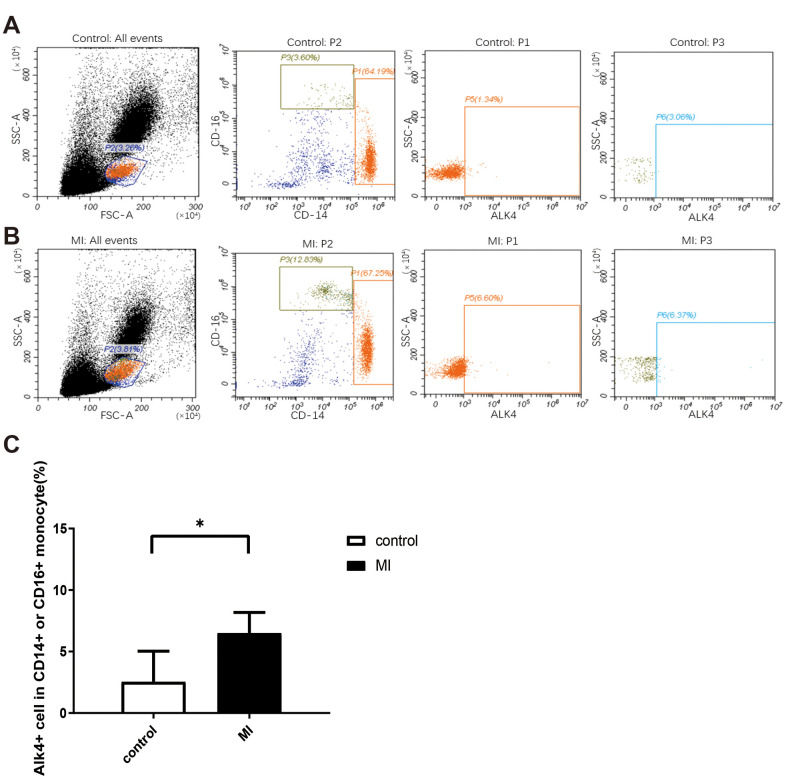
**Upregulated ALK4 expression in peripheral blood mononuclear cells of AMI patients.** Flow cytometry analysis of human peripheral blood mononuclear cells were showed. PBMCs were isolated from acute myocardial infarction patient (**A**) and healthy person (**B**). ALK4, CD16 and CD14 were detected (n=9, each group). (**C**) Bar graph presenting the number of ALK4(+) cells in CD16(+) or CD14(+) monocytes in healthy controls and AMI patients, p=0.0011. *P < 0.05 vs. healthy controls. AMI, acute myocardial infarction.

We analyzed the percentage of CD14^+^ or CD16^+^ subsets using flow cytometry in healthy volunteers and MI patients (n=9 in each group). In human blood samples, ALK4 gene expression elevated progressively in circulating monocytes among patients suffering from MI. ALK4 was found increased mostly in CD14^+^ or CD16^+^ labelled mononuclear cells in MI patients compared with that in the healthy individuals (Figure 1).

### Reduced cardiac inflammation response after myocardial injury in ALK4^+/-^ mice

**Figure 2:**
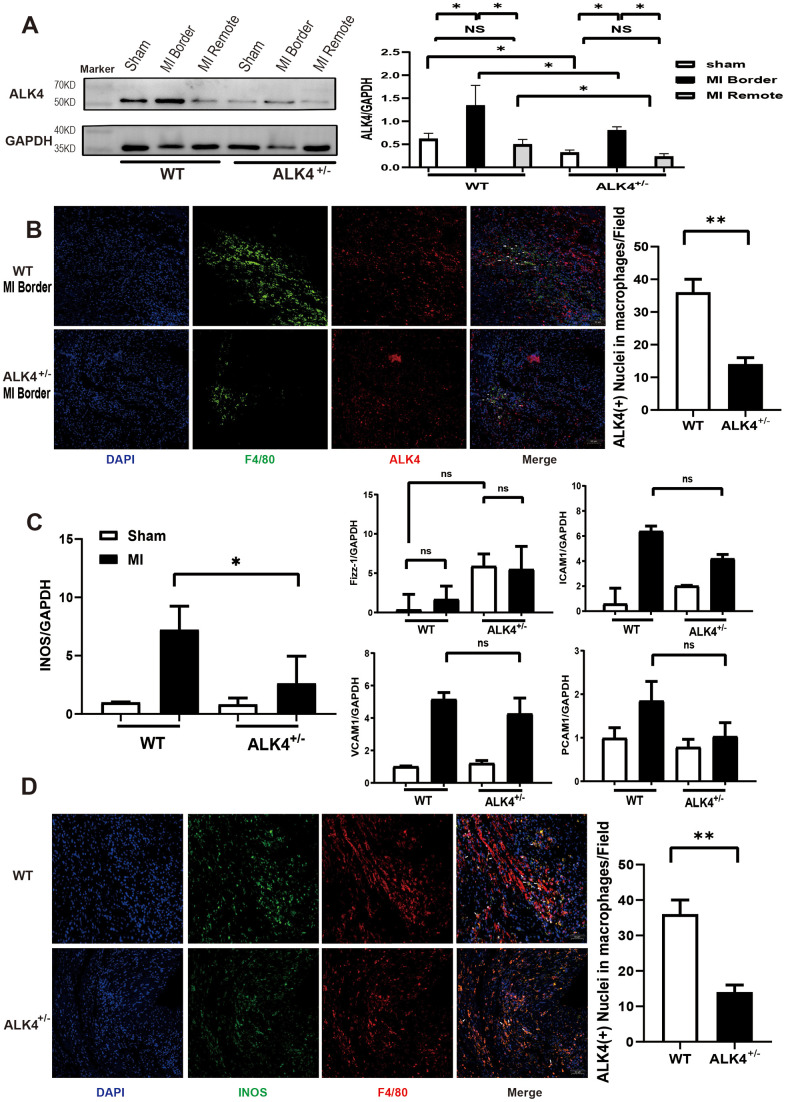
**ALK4 haplodeficency reduces cardiac inflammation after myocardial injury.** (**A**) ALK4 protein expression in WT and ALK4^+/-^ mice in 4 groups (n=4 for each). (**B**) ALK4 protein expression in macrophages in WT and ALK4^+/-^ mice on the 3rd day post-MI (n=5). (**C**) The mRNA expression of iNOS, Fizz-1, ICAM1, VCAM1 and PECAM1 were showed. (**D**) INOS expression in WT and ALK4^+/-^ mice on the 3^rd^ day post-MI (n=5). *P < 0.05. **P < 0.01. NS: not significant.

Due to the early embryonic lethality of the homozygous mice (ALK4^-/-^) [17], only ALK4^+/-^ mice were available for the present study. WT littermates and ALK4^+/-^ mice were used to establish the MI model. As proved in our previous research, ALK4 expression was elevated in heart tissues gradually in the murine model of MI. On the 7^th^ day after MI, ALK4 expression was significantly elevated in the border zone of the infarcted heart [15]. In this study, we found that ALK4 protein levels significantly increased in the border zone while showing no change in the remote area of the infarcted hearts on the 3^rd^ day after MI (Figure 2A, 2B). It implied that ALK4 played a key role in the early phase of myocardial injury. On the 3^rd^ day after MI, ALK4^+/-^ mice showed significantly reduced ALK4 upregulation and reduced macrophage infiltration in the border zone compared to WT littermates (Figure 2A, 2B). Meanwhile, co-staining of ALK4 and F4/80 revealed that MI-induced macrophages expressed ALK4 (Figure 2B). The above results provided evidence for the potential involvement of macrophages-expressed ALK4 in the inflammatory phase of MI.

We further detected the expression of M1-type, M2-type macrophages and adhesion factors on the 3^rd^ day after MI. A significantly attenuated iNOS mRNA expression was found in ALK4^+/-^ mice (Figure 2C). There was no difference in the Fizz-1 mRNA expression between the sham group and the MI group in both ALK4^+/-^ mice and WT littermates, but the baseline of Fizz-1 mRNA expression was higher in ALK4^+/-^ mice than that in WT littermates. The expression of ICAM1, VCAM1, and PECAM1 mRNA increased after MI, but their expressions showed no statistical significance (Figure 2C). Furthermore, pro-inflammatory macrophages (F4/80^+^iNOS^+^) were less observed in ALK4^+/-^ mice than those in WT littermates (Figure 2D).

**Figure 3:**
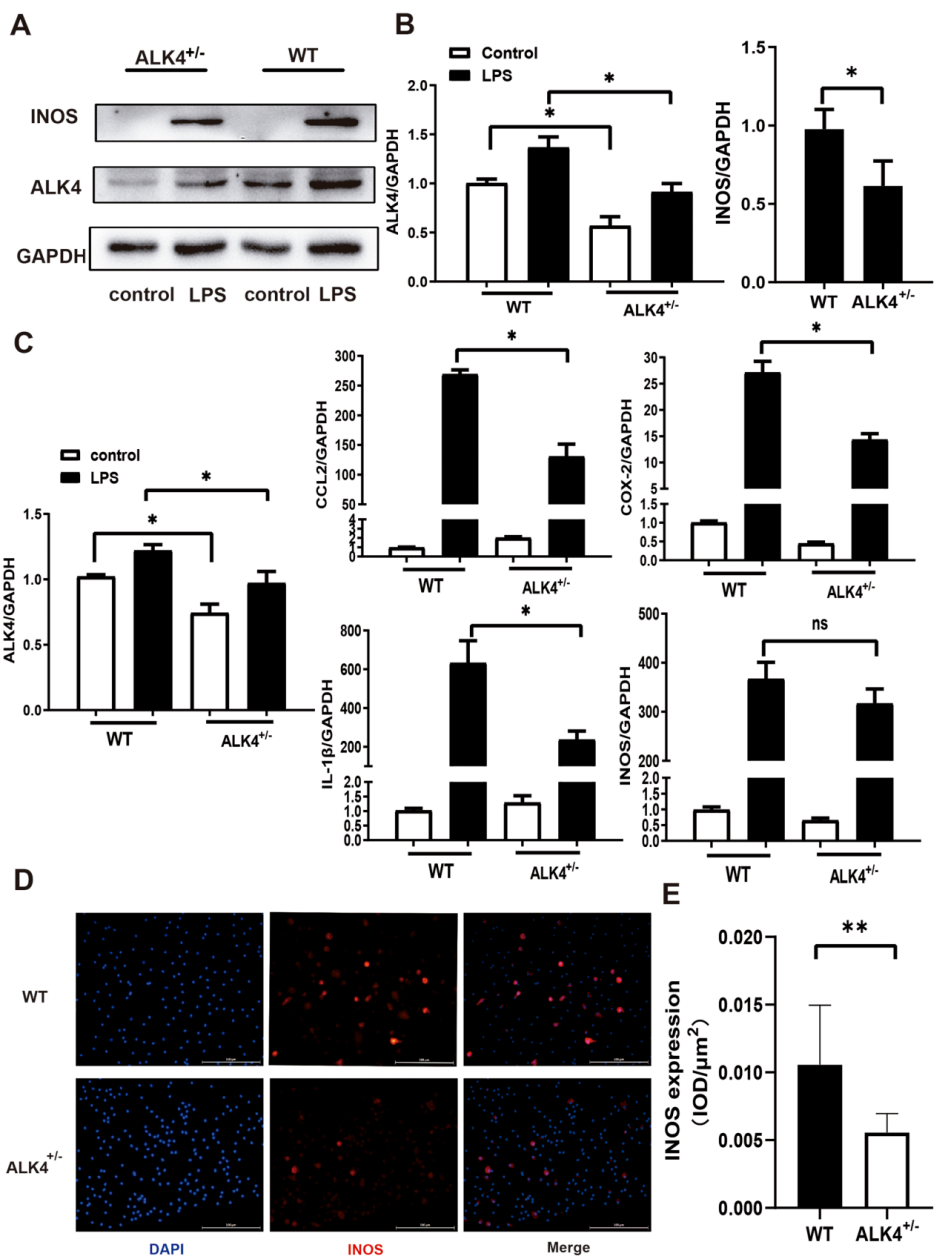
**ALK4 haplodeficency reduces pro-inflammation response.** (**A**, **B**) ALK4 protein and iNOS expression in BMDM from WT and ALK4^+/-^ mice in 4 groups (n=4 for each). (**C**) The mRNA expression of ALK4, iNOS, CCL2, IL-1β and COX-2 were detected in 4 groups (n=4 for each). (**D**, **E**) iNOS expression in LPS-stimulation BMDM from WT and ALK4^+/-^ mice in immunofluorescence. * p<0.05. ** p<0.01. NS: not significant.

Next, bone marrow-derived macrophages (BMDM) from ALK4^+/-^ mice and WT littermates were obtained as mentioned previously. After LPS stimulation under hypoxia condition for 24 hours, ALK4 expression was increased in BMDM (Figure 3A, 3B), and the expression of pro-inflammatory classical-MФ/ (M1) markers, (such as iNOS, COX2, IL-1β, and CCL2) were increased significantly. Reduced pro-inflammatory cytokines expression were found in macrophages of ALK4^+/-^ mice compared to those in WT littermates (Figure 3C–3E). These results showed that ALK4 deficiency could somehow inhibit chemotaxis and reduced pro-inflammatory classical-MФ/ (M1) after myocardial injury.

### Impact of ALK4 haplodeficiency on cardiac function and electrophysiological property after acute myocardial injury in mice

**Figure 4:**
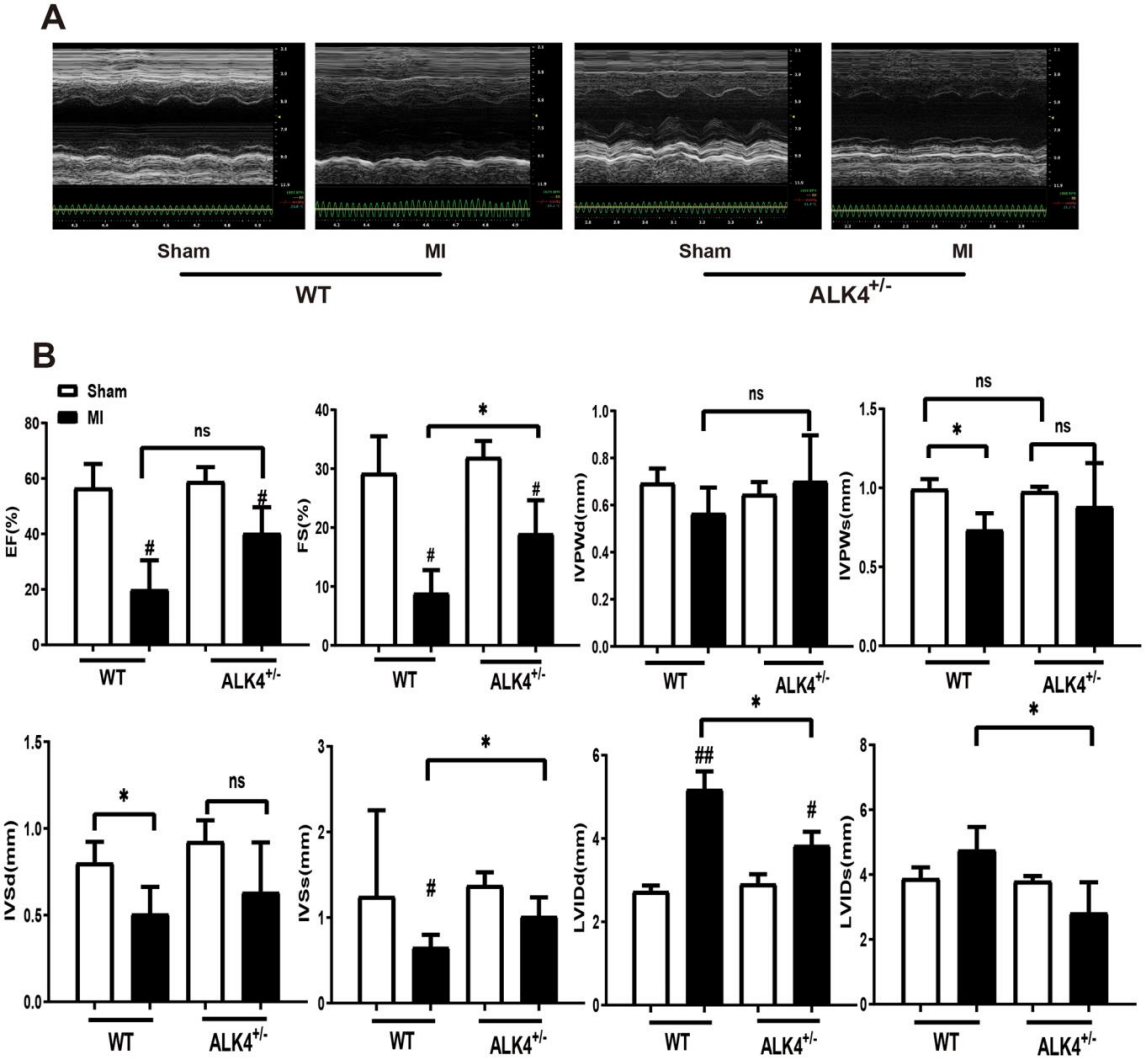
**ALK4 haplodeficiency ameliorates MI-induced cardiac dysfunction.** (**A**) M-mode echocardiograms obtained on the 3^rd^ day post-MI. (**B**) Quantitative analysis of %EF, %FS, IVPW, IVS and LVID in both mice (n > 4 for each). *P < 0.05 compared with WT sham. #P < 0.05 compared with WT after MI. NS: not significant.

The baseline echocardiographic indexes of cardiac function between the WT littermates and ALK4^+/-^ mice showed no difference (Supplementary Figure 1A, 1B). Both %EF and %FS decreased in ALK4^+/-^ mice group and WT mice group after MI, however, ALK4^+/-^ mice showed less deteriorated cardiac function on the 3^rd^ day after MI (Figure 4A, 4B). Furthermore, ALK4^+/-^ mice showed less-dilated left ventricular dimensions (LVIDd) and less-systolic left ventricular dimensions (LVIDs) (Figure 4B). The heart rate showed no difference in both ALK4^+/-^ mice and WT littermates (Supplementary Figure 1C).

The statistics of physiological parameters in each group were shown in Table 1. Both the lung weight/body weight (LW/BW) and heart weight/body weight (HW/BW) at baseline and on the 3^rd^ day after MI showed an improved trend in ALK4^+/-^ mice compared to those in WT littermates, even though there was no significant difference. We examined the infarcted area after ligation of coronary vessels for 24 hours and the level of cardiac fibrosis on the 3^rd^ day after MI to eliminate the effect of fibrosis on cardiac function. ALK4 haplodeficiency neither affected the infarcted area nor the level of cardiac fibrosis (Supplementary Figure 1D, 1E).

**Table 1:** Biometric and histological data at baseline, 3 days post-MI.

		**Sham**			**3 days post-MI**	
		WT(n=7)	ALK4+/-(n=5)		WT(n=6)	ALK4+/-(n=6)
HW/BW (mg/g)		4.13±0.84	4.38±0.66		5.58±0.95**	5.17±0.94*
Lung/BW (mg/g)		5.06±1.21	4.63±1.30		5.53±1.40	4.78±0.94

*HW/BW* heart weight/body weight, *Lung/BW* lung weight/body weight.

**p<0.01 WT post-MI vs. WT Sham.

*p<0.05 ALK4^+/-^ post-MI vs. WT Sham.

**Figure 5:**
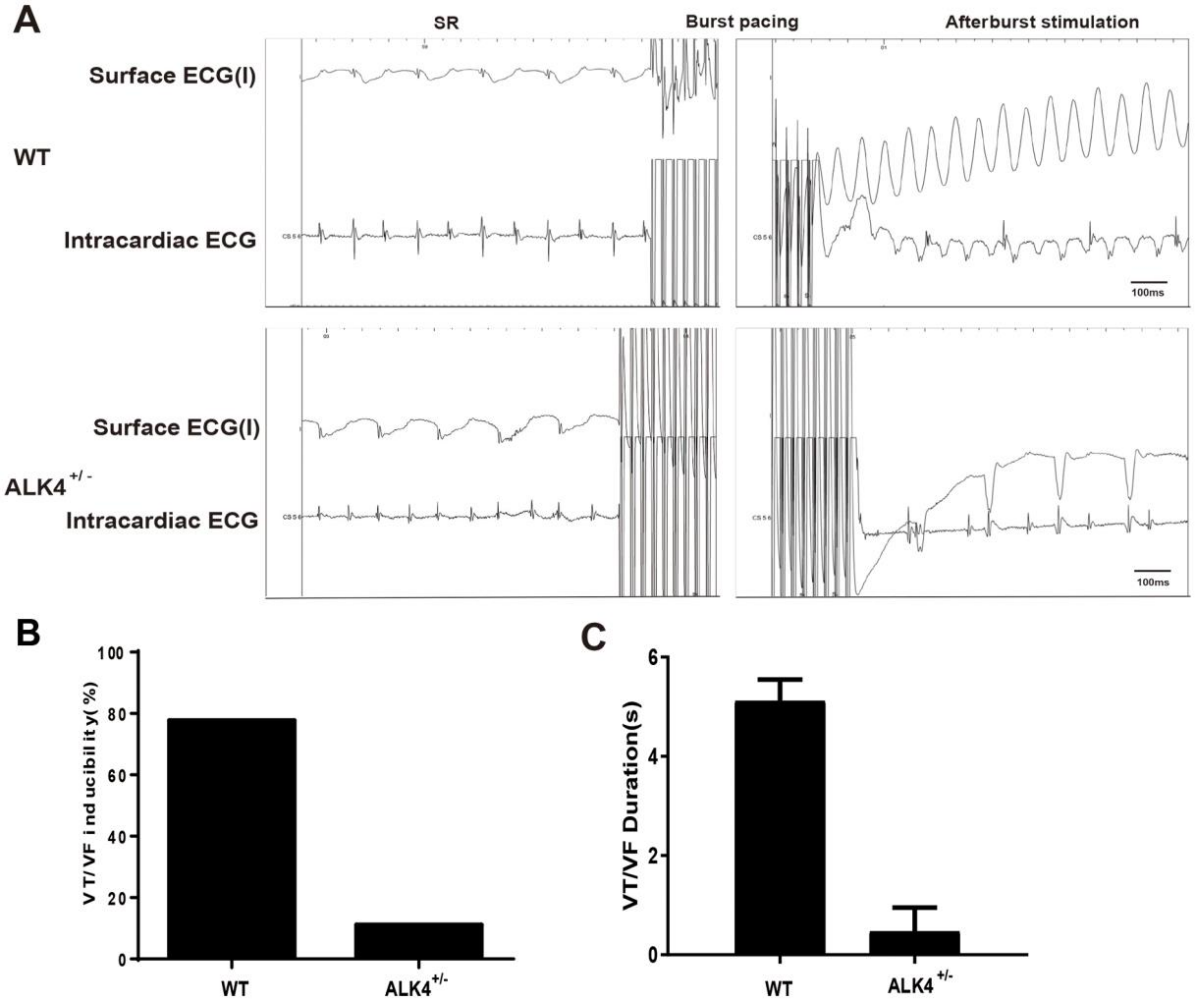
**ALK4 haplodeficiency ameliorates pacing-induced ventricular arrhythmias in MI mice.** (**A**) Representative surface ECGs and intracardiac images of intracardiac electrical stimulation of WT and ALK4^+/-^ MI mice. (**B**, **C**) VT/VF inducibility (**B**) and duration (**C**) of WT and ALK4^+/-^ MI mice (n≥4).

Next, the *in-vivo* electrophysiological (EP) study was performed. None of the mice showed spontaneous arrhythmias. However, WT littermates showed increased vulnerability to ventricular tachycardia/ventricular fibrillation (VT/VF) (n≥4, Figure 5B) and increased VT/VF duration compared to ALK4^+/-^ mice on the 3^rd^ day after MI (Figure 5C). After burst stimulation of intracardiac electrical stimulation, VF occurred in WT mice while sinus rhythm was still maintained in the ALK4^+/-^ mice on the 3^rd^ day post-MI (Figure 5A). These results indicated that ALK4 haplodeficiency plays a protective role in the inflammatory phase of MI and attenuates the vulnerability of VT/VF during the *in-vivo* EP study.

### ALK4 haplodeficiency promotes the cardiac remodeling after myocardial injury in mice

**Figure 6:**
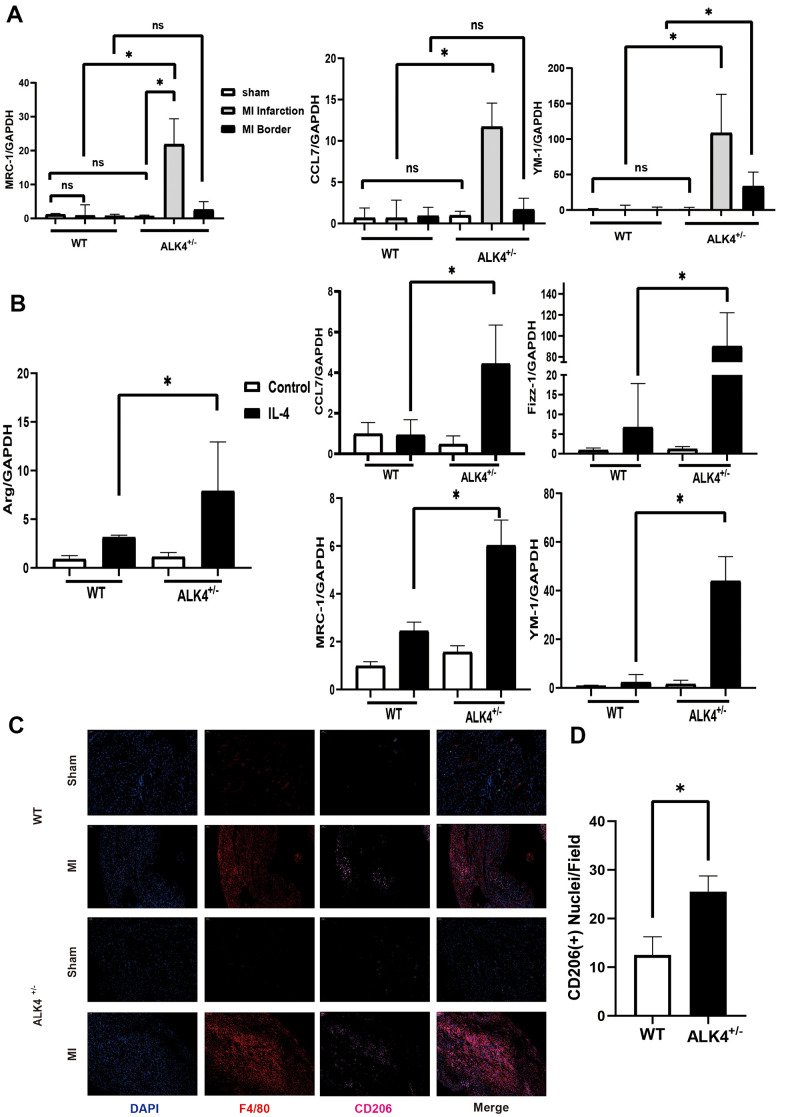
**ALK4 haplodeficency promotes anti-inflammation response.** (**A**) The mRNA expression of MRC-1, CCL7 and YM-1 were detected in WT and ALK4+/- mice on the 7th day post-MI (n=4 for each). (**B**) The mRNA expression of Arg, CCL7, Fizz-1, MRC-1 and YM-1 were detected in bone marrow derived macrophages from WT and ALK4^+/-^ mice stimulated by IL-4 in 4 groups (n=4 for each). (**C**) CD206 expression in WT and ALK4^+/-^ mice on the 7th day post-MI in immunofluorescence (n=4 for each). (**D**) The quantitative analysis of the CD206 expression in the MI group. * p<0.05. NS: not significant.

The expression level of M2 macrophages markers (Mrc-1, YM-1, CD206, and CCL7) in the infarction and border zones on the 7^th^ day after MI were detected to explore the ability of cardiac repair. M2 macrophages increased significantly in the infarction zone compared to those in the border zone, while more M2 macrophages were showed in the hearts of ALK4^+/-^ mice than those in WT mice (Figure 6A). Immunofluorescence results also showed that the expression of CD206 was significantly increased in ALK4^+/-^ mice after MI (Figure 6C, 6D). Next, BMDM from ALK4^+/-^ mice and WT littermates were obtained. After 24h-stimulation by IL-4, the mRNA expression of anti-inflammatory classical-MФ/ (M2), such as Arg, CCL7, Mrc-1, Fizz-1, and YM-1, were significantly increased. Moreover, increased anti-inflammatory cytokines were observed in ALK4^+/-^ macrophages compared to those in WT littermates’ macrophages (Figure 6B). Taken together, ALK4 haplodeficiency might induce the phenotype switch of pro-inflammatory classical-MФ/ (M1) macrophages towards anti-inflammatory classical-MФ/ (M2) macrophages, and finally promoted cardiac repair after myocardial injury.

### ALK4 haplodeficiency attenuates activation of the Smad2/3 pathway

**Figure 7:**
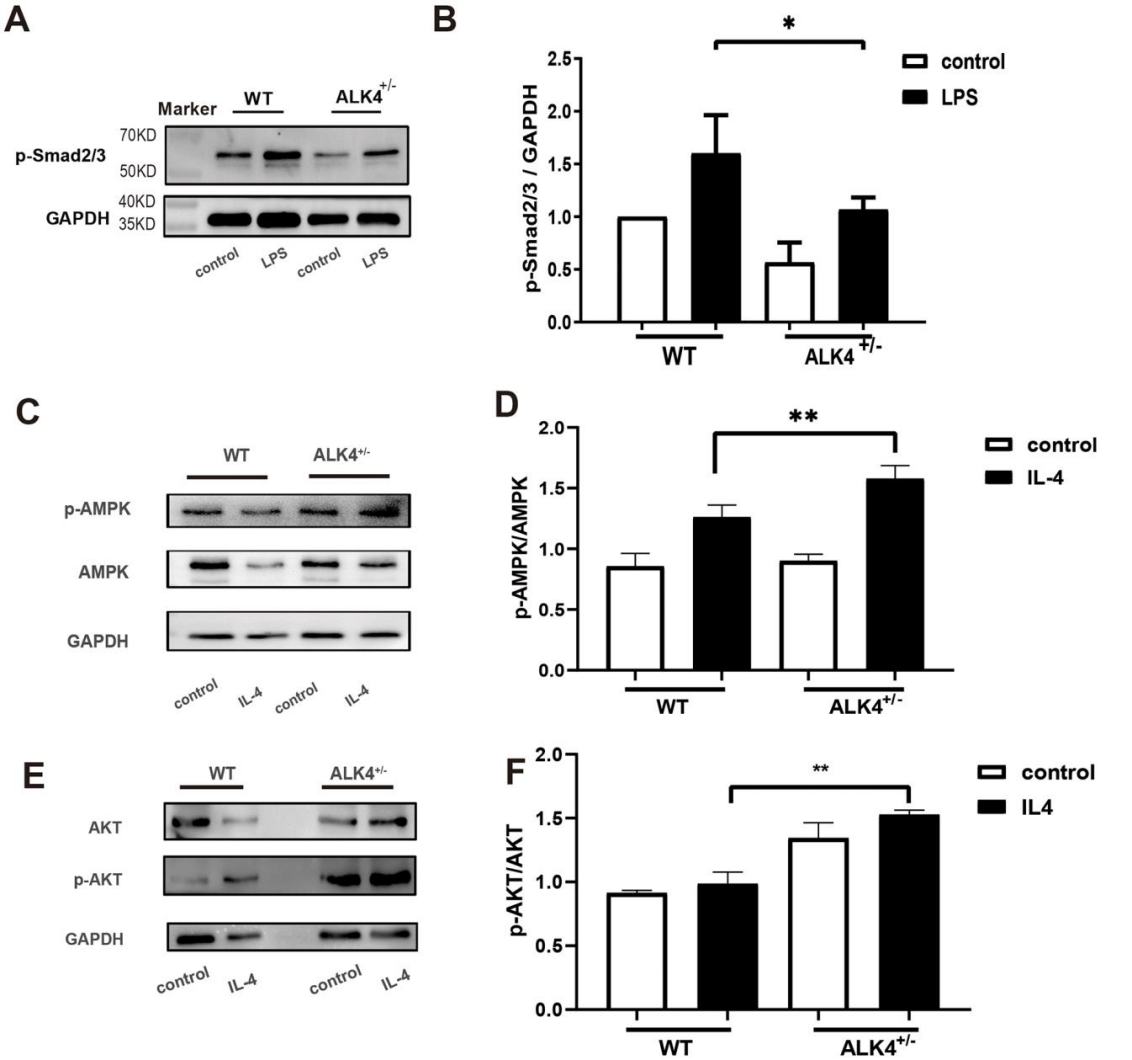
**ALK4 haplodeficiency attenuates the activation of the Smad2/3 pathway and promotes activation of the AMPK pathway in macrophages.** (**A**, **B**) Smad2/3 phosphorylation expression in macrophages deriving from the bone marrows of WT and ALK4^+/-^ mice in 4 groups (n=4 for each). (**C**, **D**) AMPK expression in bone marrow derived macrophages from WT and ALK4^+/-^ mice stimulated by IL-4 in 4 groups (n=4 for each). (**E**, **F**) ATK expression in bone marrow derived macrophages from WT and ALK4^+/-^ mice stimulated by IL-4 in 4 groups (n=4 for each). * p<0.05.

To explicate the potential molecular mechanism regarding the function of ALK4 in the inflammatory phase of MI, we explored the Smads pathways in BMDM of each group. Compared with WT littermates, ALK4^+/-^ mice significantly blunted LPS-induced upregulation of p-Smad2/3 in macrophages (Figure 7A, 7B). The data suggested that ALK4 haplodeficiency exerted beneficial effects on inflammatory response and reduced the occurrence of ventricular arrhythmia post-MI by decreasing Smad2/3 phosphorylation.

### ALK4 haplodeficiency enhances activation of the AMPK pathway

AMPK is most widely known for its role as the energy state sensor. AMPK exerts cardiovascular protective function due to its critical function in metabolic homeostasis [18]. To explore the molecular mechanism of ALK4 in the process of cardiac repair, we detected the expression of AMPK pathway in IL-4-stimulated BMDM. Compared with WT littermates, ALK4^+/-^ mice significantly aggregated IL-4-induced upregulation of AMPK and AKT in macrophages (Figure 7C–7F). The data suggested that ALK4 haplodeficiency exerted beneficial effects on inflammatory response and promoted cardiac repair post-MI by increasing AMPK phosphorylation.

## DISCUSSION

In this study, it was the first time to demonstrate the role of ALK4 in inflammation response after acute MI. Inflammation responses influence the functional and structural changes of the left ventricle, and the defective resolution of inflammation causes increased mortality in MI patients [19, 20]. The potential protective effects of ALK4 haplodeficiency on inflammation post-MI were examined in the MI mouse model. Co-staining of ALK4 and F4/80 revealed that expression of ALK4 was upregulated in the border zone of the infarcted heart after MI, which indicated the potential involvement of macrophages-expressed ALK4 in the inflammatory phase of MI.

Inflammatory response is a necessary process in the formation and development of MI-induced cardiac fibrosis, and macrophages are one of the main inflammatory cells involved in the inflammatory response. Our previous study demonstrated that ALK4 haplodeficiency attenuated cardiac fibrosis on the 28^th^ day after MI [15]. However, previous study just considered the hypoxia-induced changes of cardiac fibroblasts, the role of inflammatory response in the process of MI-induced cardiac fibrosis was not taken into account. In this study, we established the acute MI model in both ALK4^+/-^ mice and WT littermates and observed the inflammatory responses on the 3^rd^ day and 7^th^ day after MI. Our study firstly demonstrated that ALK4 haplodeficiency did not affect the infarcted area after 24h-ligation of coronary vessel and fibrosis level on the 3^rd^ day after MI. Previous studies demonstrated that increased inflammation and the resolution of inflammation following acute MI contribute to ventricular remodeling [21, 22]. Other studies demonstrated that activin A could influence the macrophages’ function as a pro-inflammatory factor [16, 23, 24]. ALK4 acts as the receptor of activin A, so we speculated that ALK4 expression might also change in macrophages. This study revealed that the effect of ALK4 haplodeficiency on inflammation response in post-MI stage was mainly attributed to the following aspects: (1) reducing the recruitment of inflammatory cells, (2) reducing the secreting of classical-MФ(M1), (3) promoting the secreting of alternative-MФ(M2), (4) alleviating the pacing-induced ventricular arrhythmias.

MI is associated with structural changes of the ventricle, mainly including ventricular dilation and decreased LVEF and %FS. MI can make electrophysiological properties of the ventricular myocytes changed as well. In all, compared with WT littermates, ALK4^+/-^ mice showed a remarkable reduction in the incidence and duration of VF/VT episodes in hypertrophied hearts. The discrepancy in the incidence might be explained by the existence of discrepant histologically detectable inflammation. The change corresponds to the suppression of VF/VT. Our results demonstrated that ALK4 haplodeficiency improved the arrhythmogenic substrate in the ventricular by reversing electrophysiological remodeling and inflammation, and finally attenuated vulnerability to VF/VT in the MI mouse model. These results suggested that a specific inhibitor of ALK4 might be a promising target for the upstream prevention in the inflammatory phase of MI. However, this study still had some limitations. The interplay between ALK4 and the transition of M1and M2 macrophages in MI remains to be explored.

Mechanistically, as the downstream part of the ALK4, Smad2/3 and ERK/CREB pathways have been demonstrated to contribute to regulating the process of inflammation and the expression of cytokines [16, 23, 24]. But this study did not directly prove the role of haplodeficient ALK4 in the pathogenesis of inflammation in a post-MI heart. Our study found that the Smad2/3 pathway contributed to regulate the inflammation response of macrophages. A recent study has found Smad3 critically regulates the function of infarct macrophages and protects the infarcted heart from adverse remodeling [25]. These studies confirmed that ALK4 could affect the macrophages by activating Smad3 pathway. The mechanism between ALK4 and Smads needs to be further studied. In addition to the Samd2/3 pathway, ALK4 was also implicated in regulating AMPK pathways associated with M2 macrophages. In our study, we found that ALK4 haplodeficiency promotes the secretion of M2 macrophage by upregulation of AMPK pathways. Meanwhile, our studies focus on the phenotype shift of M1 towards M2, implying that ALK4 could be a promising therapeutic target for the inflammation response in the early phase of MI. This comprehensive understanding of the protective role of haplodeficient ALK4 in the pathogenesis of inflammation in the early phase of MI is expected to foster the development of improved pharmacological therapeutic approaches.

## MATERIALS AND METHODS

### Ethical statement

The study was approved by the ethics committee of Shanghai Xinhua Hospital affiliated to Shanghai Jiao Tong University School of Medicine. All animal procedures were conducted in compliance with the National Institutes of Health Guidelines for the care and use of laboratory animals.

### Human blood sample

Written consent was obtained from all enrolled participants. Human peripheral blood was collected from non-ST segment elevation MI patients on the 3^rd^ post-MI before percutaneous transluminal coronary intervention (PCI) in Xinhua Hospital affiliated to Shanghai Jiao Tong University School of Medicine. The control group enrolls healthy volunteers with no cardiac disease. The investigation was confirmed to the principles outlined in the Declaration of Helsinki.

### Flow cytometry analysis

2ml heparin-treated blood was used to isolate the human peripheral blood mononuclear cells (PBMCs) by density gradient centrifugation Ficoll Paque (GE Healthcare, Piscataway, NJ, USA). FITC-conjugated anti-ALK4 antibody (R&D), PE-conjugated anti-CD16 antibody (Biolegend), and APC-conjugated anti-CD14 antibody (Biolegend) were used for flow cytometry. After the PBMCs were incubated with antibodies, washed, and fixed, the samples were analyzed by Flow software (CytExpert) and flow cytometry (CytoFLEX, Beckman Coulter, Inc. Brea, CA, USA).

### ALK4 haplodeficiency mice and MI model

We used the CRISPR-Cas9 technology to generate the ALK4 haplodeficiency mice (ALK4^+/-^) as mentioned previously [26]. ALK4 haplodeficiency (ALK4^+/-^) mice and wild-type (WT) littermates were all generated in the C57BL/6J background. Eight-week-old male mice were randomly divided into 4 groups (n=6 in each group): (1) WT littermates undergoing sham operation; (2) WT littermates undergoing MI operation; (3) ALK4^+/-^ mice undergoing sham operation; (4) ALK4^+/-^ mice undergoing MI operation. MI was performed by ligation the branch of the left anterior descending of the coronary artery permanently, as described previously [27]. Briefly, mice were anesthetized with 2% isoflurane inhalation and aerated by orotracheal intubation. After left thoracotomy, 6-0 silk sutures were used to ligate the coronary artery. When the artery was ligated successfully, the anterior wall of the ligated heart would turn pale immediately. The mice in the sham operation group were subjected to a similar procedure without ligation. 2% Evans blue was perfused to the heart 24 h after MI to determine an area at risk(AAR) around the infarct zone.

### Echocardiography

Before the operation and on the 3^rd^ day after the operation, mice were anesthetized with 2% isoflurane inhalation. The ejection fraction (EF), fractional shortening (FS), LV end-systolic (LVIDs), and end-diastolic (LVIDd) dimensions were measured from the M-mode images of a 15 MHz transducer echocardiography. The information about the genotype of the mice was blinded to the sonographer.

### Intracardiac electrophysiological recordings

Intracardiac electrophysiological (EP) studies were undergoing in all ALK4^+/-^ mice and WT littermates by a blinded operator. Mice were anesthetized by 2% isoflurane inhalation and placed on a heated pad at 37° C to maintain body temperature. The inducibility of ventricular tachycardia/ ventricular fibrillation (VT/VF) was assessed by *in-vivo* intracardiac programmed electrical stimulation. Both surface electrocardiogram (ECG) and intracardiac electrogram were recorded synchronously and analyzed by the LabChart Software 7.0. A 1.1-F electrode catheter (Scisense, London, ON, Canada) was advanced through the right jugular vein into the right ventricular apex on the 3^rd^ day post-MI [28, 29]. For burst pacing, a pacing train of 30 stimuli (S1) was delivered at cycle lengths of 30 ms. For extra-stimulus pacing, 3 premature stimuli were delivered following 7 paced beats at a basal cycle length of 100 ms. After echocardiography and the intracardiac electrophysiological investigation had been performed, mice were anesthetized with sodium phenobarbital (100 mg/kg) by abdominal injection and euthanized by swift decapitation according to NIH guidelines. Then the heart and tibia were extracted separately.

### Isolation of bone marrow-derived macrophages

Bone tibia and femurs were collected from the ALK4^+/-^ mice and WT littermates in the ice-cold PBS and stripped from muscles. After placing the stripped bones in DMEM/High Glucose and washed again after the ends cut off and the inner bone marrow was flushed out with a 25 G syringe filled with cold DMEM/High Glucose with 10% fetal calf serum and 100 U/ml penicillin/streptomycin. The flushing of single cell suspension from all the bones was done by passing the suspension through a 100 μm cell strainer. Cells were spun down at 1000 rpm after effected by Red Blood CELL Lysis Buffer (Beyotime) and placed in bacterial plates in DMEM/High Glucose containing 10% fetal calf serum and 100 U/ml penicillin/streptomycin with 10% M-CSF (Sigma) for culture and differentiation. Differentiation for approximately 8-10 days, the medium was added or replaced every 3-5 days and experiments were performed after it. Cells were counted and cultured in 6-wells plates and divided into two groups, namely the control group and the LPS group. The Control group was culture for 24 h under hypoxia condition, and the LPS group was stimulated with LPS (0.6 ng/ml, Sigma) for 24h under hypoxia condition. For hypoxia condition, cells were cultured in an incubator filled with 1% O_2_. While cells were also cultured in 6-wells plates and divided into a control group and IL-4 group for 24 h under normoxia condition. IL-4 group was stimulated with IL-4 (20 ng/ml, Abcam).

### Histology and immunofluorescence

Mice were anesthetized with sodium pentobarbital (100 mg/kg, i.p.) by abdominal injection on the 3^rd^ day and the 7^th^ day after MI or sham surgery. Harvested hearts and cultured cells were fixed with 4% paraformaldehyde, embedded in paraffin, and transversely sectioned at 5 μm thickness. Left ventricular sections were incubated with primary antibodies against ALK4 (Abcam), F4/80 (Abcam), iNOS (Abcam) and CD206(Abcam), and subsequently with fluorescent secondary antibodies Alexa Fluor 488 (Invitrogen), Alexa Fluor 555 (Invitrogen). Cells were stained with DAPI for nuclei. Scanner (3D Histech) was used for analyzing, as well as Image-Pro Plus 6.0 software (Media Cybemetics) and Caseviewer software (3D Histech). More than 2 sections of the left ventricles from each mouse samples were randomly selected to quantify the number of macrophages. In each well of plates, two images were analyzed.

### Western blotting and quantitative real-time PCR

Heart tissues and cultured cells were dissolved in SDS-sample buffer, sonicated, subjected to SDS-PAGE, and transferred to PVDF membranes as described previously [30]. Then put the PVDF membranes at 4° C incubated overnight with the primary antibodies against including ALK4 (Abcam), iNOS (Abcam), Smad2/3(Abcam), p-MAPK(CST), MAPK(CST), AKT(CST), p-AKT(CST). The dilution factor for the iNOS, Smad2/3, p-MAPK, MAPK, p-AKT, and AKT antibodies was 1:1000, while ALK4 antibody was 1:500. Membranes were incubated with peroxidase-conjugated secondary antibodies for 2 h at room temperature after washed. Gel Imaging System (Tanon) was used to image and AlphaView software was used to analyze Western blots.

Total RNA was extracted from the heart tissues and cultured cells by using TRIzol (Takara). 1000 ng total RNA was reversely transcribed into cDNA using the Prime-Script™RT reagent kit (Takara). Using SYBR green (Takara) to performed qRT-PCR and normalized to GAPDH expression to describe transcript levels of ALK4 (forward primer CGGTCTTGGTTCAGGGAAG, reverse primer CTGTGTCCAGGTGCCATTATC), iNOS (forward primer CACAAGCTGGCTCGCTTTGC, reverse primer TGGCCCTGCTCCCCGTGGAGC), COX-2(forward primer ACTCACTCAGTTTGTTGAGTCATTC, reverse primer TTTGATTAGTACTGTAGGGTTAATG), CCL2 (forward primer GCCAACTCTCACTGAAGCC, reverse primer GCTGGTGAATGAGTAGCAGC), TGF-β(forward primer CGCTGACATCTATGCAATGG, reverse primer CAACCGATGGATCAGAAGGT), Fizz-1 (forward primer CCAATCCAGCTAACTATCCCTCC, reverse primer CCAGTCAACGAGTAAGCACAG), ICAM1(forward primer GTGATGCTCAGGTATCCATCCA, reverse primer CACAGTTCTCAAAGCACAGCG), VCAM1(forward primer CCGGCATATACGAGTGTGAA, reverse primer GATGCGCAGTAGAGTGCAAG), PECAM1(forward primer ACGCTGGTGCTCTATGCAAG, reverse primer TCAGTTGCTGCCCATTCATCA), Arg (forward primer GCTCAGGTGAATCGGCCTTTT, reverse primer TGGCTTGCGAGACGTAGAC), Mrc1(forward primer CTCTGTTCAGCTATTGGACGC, reverse primer TGGCACTCCCAAACATAATTTGA), YM1(forward primer CAGGTCTGGCAATTCTTCTGAA, reverse primer GTCTTGCTCATGTGTGTAAGTGA).

### Statistical analysis

Data were presented as median ± IQR. Statistical analysis was performed with the GraphPad Prism 8 and IBM SPSS Statistics 22. Mann-Whitney U test and one-way ANOVA test were used to determine statistical significance. A value of p<0.05 was considered as statistically significant.

## Supplementary Material

Supplementary Figure 1
