# Supplementary material for: Activin receptor-like kinase 4 haplodeficiency alleviates the cardiac inflammation and pacing-induced ventricular arrhythmias after myocardial infarction
Source: Aging (Albany NY). 2021 Jul 1;13(13):17473–88. doi: 10.18632/aging.203236 (PMC8312420; doi:10.18632/aging.203236)
Supplement: Supplementary Figure 1 [file aging-13-203236-s001.pdf]

SUPPLEMENTARY FIGURE

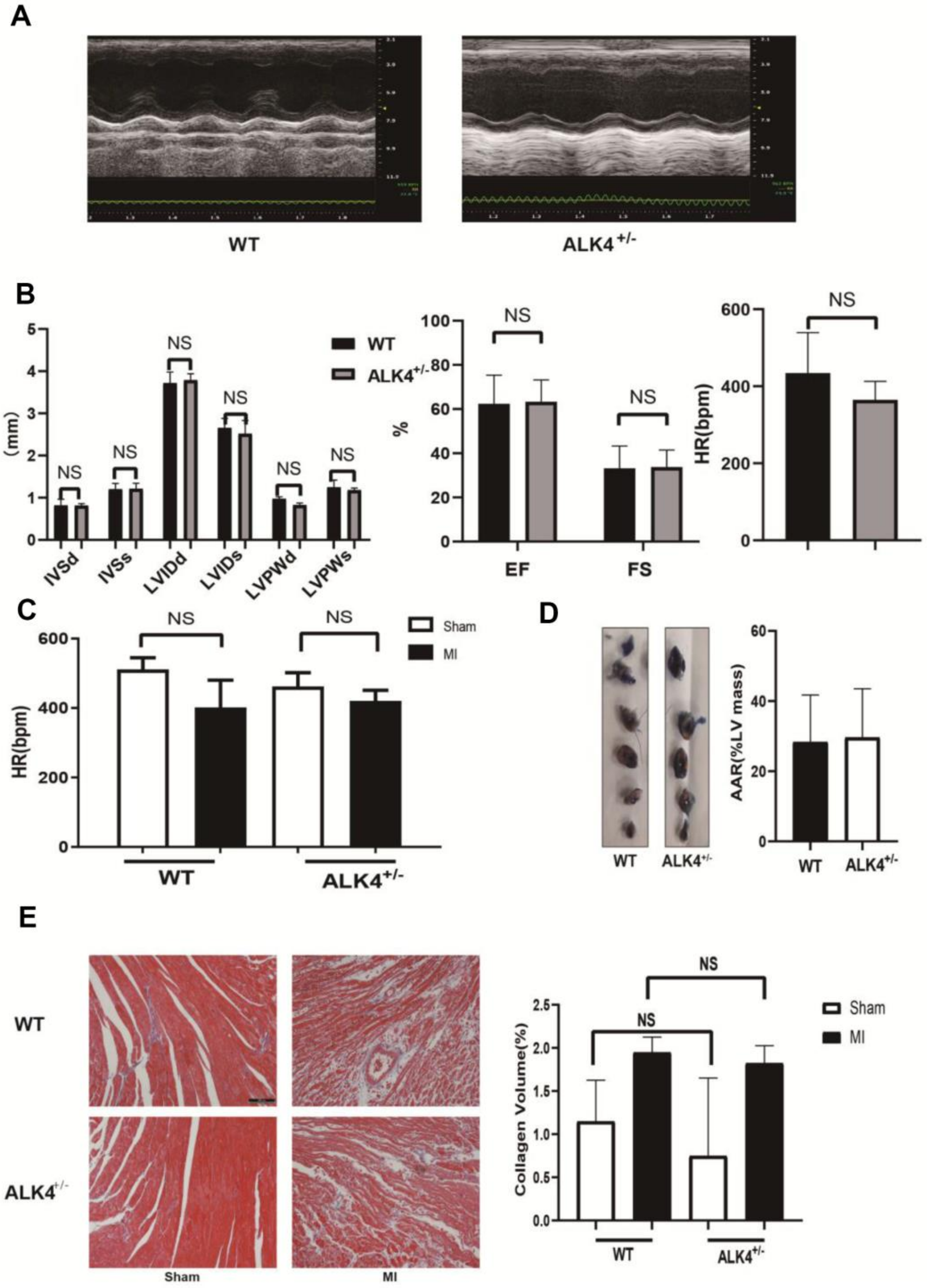

Supplementary Figure 1. (**A**) The M-mode echocardiograms obtained before MI operation in WT and ALK4+/- mice. (**B**) Quantitative analysis of %EF, %FS, HR, IVPW, IVS and LVID in both mice before operation (n>4 for each). (**C**) Quantitative analysis of HR in the sham and MI model of the ALK4+/- and WT mice. (**D**) Images and quantitative analysis of the area at risk(AAR) determined by Evans blue perfusion at 24 h after MI(n=4 for each). (**E**) Representative histological images of the Masson staining and quantitative analysis of the stained fibrotic areas of the LV in sham group and of the border zone in MI group in WT and ALK4+/- mice (n=6 for each). NS: not significant.
